# Will tobacco price increases lead more people who smoke to vape? The results from a discrete choice experiment amongst U.S. adults

**DOI:** 10.1186/s12889-023-17094-5

**Published:** 2023-11-20

**Authors:** Gilda Zarate-Gonzalez, Paul Brown, Linda D. Cameron, Anna V. Song

**Affiliations:** University of California, Merced 5200 North Lake Rd., Merced, CA 95343 USA

**Keywords:** Smoking, Vaping, Nonsmokers, Discrete choice experiments, Policy options

## Abstract

**Objective:**

To understand the extent to which people who smoke, people who vape and nonsmokers would switch between smoking cigarettes and vaping in response to policies (price increases, restrictions on nicotine, places, and information on addictiveness and/or health risks) aimed at decreasing tobacco use by people who smoke and vaping by nonsmokers.

**Design:**

A total of 525 adults aged 18 to 88 years completed a discrete choice survey of 16 choices between two smoking/vaping alternatives. Analysis was conducted using conditional logistic regression for the entire sample and stratified by nonsmokers, people who smoke, and people who vape.

**Results:**

The results suggest that most people who vape also smoke. Nonsmokers were more favorable to vaping and were concerned about long-term health risks and cost associated with vaping. Marginal analysis suggests that price increases will have only modest success in moving people who smoke to start vaping or encouraging people who vape to vape rather than use cigarettes. Nonsmokers are not very sensitive to price changes but are sensitive to information about health impacts.

**Conclusions:**

Findings indicate that increasing the price of cigarettes would lead to a limited increase in the probability of people who smoke switch to vaping. The study advances our understanding of the views of current nonsmokers toward cigarettes and vaping, suggesting that price increases and increased knowledge of addiction would likely deter nonsmokers from vaping. Changing the amount of nicotine associated with smoking would increase the probability of vaping slightly and have little impact on nonsmokers or vaping preferences, but the most significant change would come from increasing the perceptions of the risk of smoking.

## What is already known on this topic

Discrete choice experiments have been used to identify the strength of preferences in tobacco products use (e.g., cigarettes, e-cigarettes, or waterpipes) in a variety of situations, including the impact of price changes on demand for cigarettes, health warnings, and restrictions on flavored products by people who smoke and people who vape. 

## What this study adds

This study examines the extent to which *people who smoke, and people who vap*e will switch between products when relative prices are changed and other restrictions, including novel attributes, are in place, and the likely impact of these policies on the perceptions of *nonsmokers*. 

## How this study might affect research, practice or policy

This study provides information to inform policy makers and public health officials on the perceptions of people who smoke, people who vape, and nonsmokers toward smoking and vaping, and the likely impact of interventions aimed at reducing smoking and/or vaping. 

## Introduction

Tobacco cigarette smoking in the U.S. has declined in recent decades, from 21% in 2005 to 11.5% in 2021 [1, 2]. However, the increased use of e-cigarettes or vaping products has raised concerns in the public health community about an increase in the risk of cardiovascular disease, inflammation, and oxidative stress [3]. This is particularly a concern if vaping is undertaken by nonsmokers who see vaping as a safe alternative to smoking [4].

Yet vaping is also promoted as a way to decrease cigarette smokers’ dependency on tobacco and aid in cessation attempts [5]. Supporters point out that vaping produces fewer of the toxins and pollutants than cigarette smoking [6]. This has led some medical and public health bodies to support vaping as a harm reduction strategy for tobacco use/smoking (hereafter: people who smoke) [7–10]. The challenge for policy makers looking to promote vaping as a smoking cessation strategy is designing policies that make vaping more attractive to people who smoke while not increasing the attractiveness of vaping to nonsmokers. Likewise, policy makers looking to curb vaping must design policies that make vaping less attractive to nonsmokers while not increasing the attractiveness of smoking to people who vape or nonsmokers.

There are several policy options that have the potential to change smoking and vaping behavior, including changes in the absolute and relative price, restrictions on nicotine or additives, restrictions on where smoking or vaping can occur, and informational campaigns highlighting the health risks or addictiveness of the products. Evaluating the relative effectiveness of these options is often difficult due to a lack of data in practice. An alternative approach to examine the impact of policies in tobacco control research is to use a discrete choice experiment (DCE). DCEs, a form of conjoint analysis methodology, are used to identify the strength of preferences in decision contexts where little choice data is available [11]. In a DCE, participants receive a series of choices between two options (e.g., smoking or vaping), with the attributes systematically varying across the decision rounds (e.g., low price versus high price, nicotine levels that produce a “low kick or nicotine” versus “high kick or nicotine”, etc.) The analyses of these repeated choices provide estimates of the strength or importance of the attribute.

DCEs have been used to examine tobacco use, vaping, and waterpipes in a variety of situations [12–17], including the impact of price changes on demand for cigarettes [16, 18], nicotine content restriction [13], health warnings [19–22], and restrictions on flavored products [23, 24]. These studies’ results suggest that prices can have some impact on demand for cigarettes by people who smoke.

These previous studies provide information on the responsiveness of smoking or vaping to several policy options. The current study extends this research by expanding the number of policy options to include potential restrictions on the satisfaction which in this study is referred to as “*kick*” (nicotine restrictions), the *place to use* the product (restrictions where people can smoke or vape), informational campaigns that highlight the *addictiveness* and *health risks* as well as the *price* of the products. During the focus group interviews and trial participants consistently referred to nicotine levels as “kick”, and after clarification and confirmation, the term was adopted for clarity and unambiguity. The study also examines the extent to which people who smoke and people who vape will switch between products when prices and other restrictions are in place, and the likely impact of these policies on the perceptions of nonsmokers. The attributes included in the DCE include the type of product (cigarettes, e-cigs, cigars, or hookahs), and the price of the product. This study adds to the existing literature by examining the differences between people who smoke, people who vape, and nonsmokers in the strength of their preferences and the extent to which their choices are sensitive to changes in factors that could be regulated.

## Methods

### Study design and DCE development

The final experiment utilized the company Qualtrics to survey participants online. Qualtrics has been used in similar studies [11, 25–27] involving behavior choice tasks in tobacco and other behavior topics. Qualtrics provides expert panel data management to avoid responses from bots and missing data [28]. To ensure that the study had a sufficient sample size to analyze, a recruitment filter was commissioned to recruit adults who smoke cigarettes, cigars, hookahs or vape e-cigarettes. A total of 525 adults, including 200 people who use any of the cigarettes, cigars, hookahs, or e-cigarette tobacco products, participated. The sampling size was determined by examining experimental studies to select a larger sample size and have adequate power to measure differences.

Following the *International Society for Pharmacoeconomics and Outcomes Research* (ISPOR) recommendations [29], attributes and levels were developed based on existing literature and validated during face-to-face interviews and focus groups with adult people who smoke or people who vape at the time of the study’s phase. The recruitment was advertised at UC Merced via flyers among staff and students. Participants first responded to open-ended questions about the factors that they considered as important when deciding whether to smoke or vape. The feedback provided in this qualitative part of the study aided the comprehension, accuracy, and presentation of the DCE choice scenarios. The interviews were recorded, transcribed, and then analyzed by a trained member of the research team. The information and feedback were incorporated into the final survey. The results were analyzed, and the individuals involved in the pilot stated that all questions were clear and unambiguous.

**Fig. 1:**
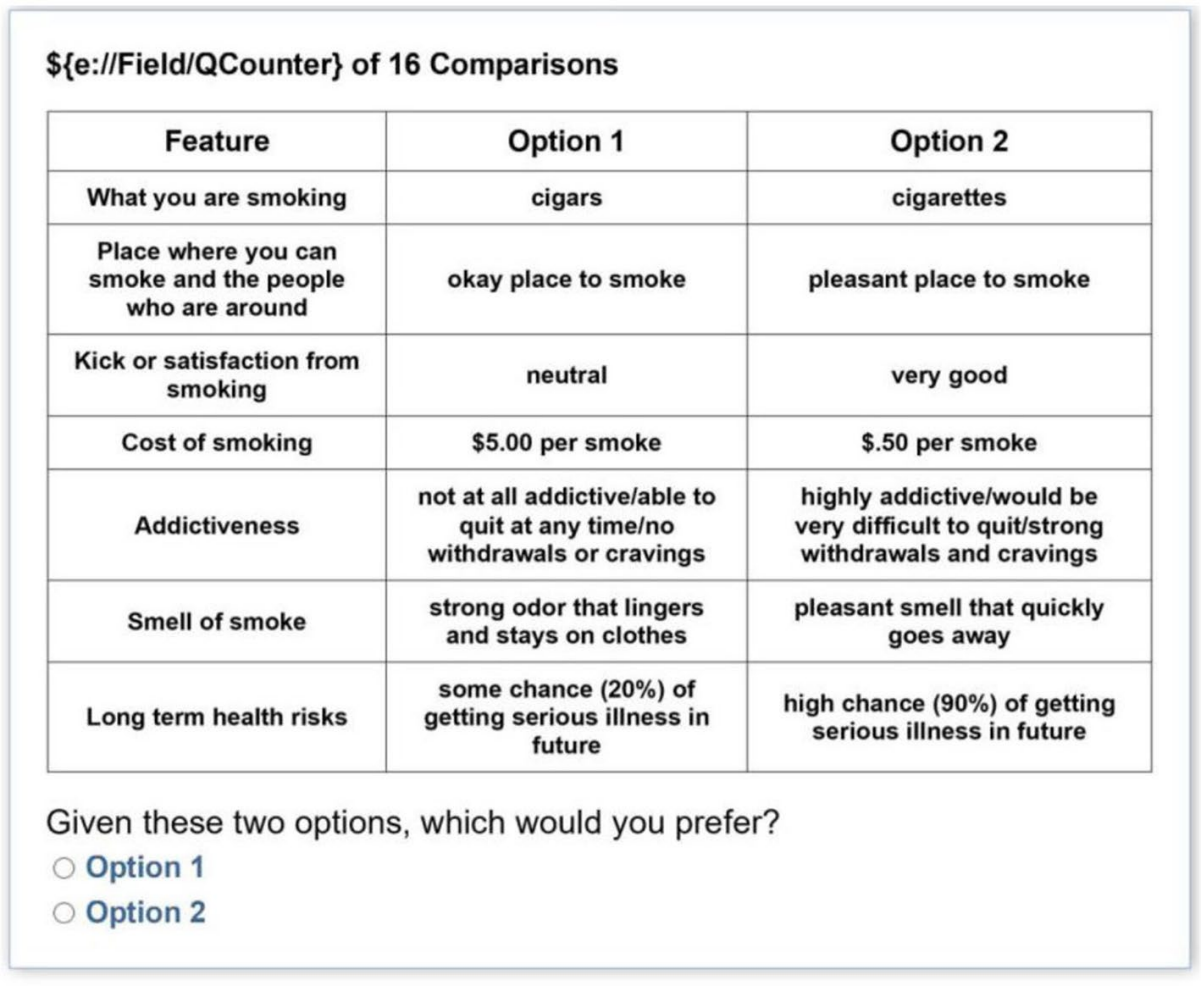
Sample choice set

The final attributes are summarized in Table 1. Each choice option was described by a set of seven attributes (see Fig. 1) and each participant was given two choices. That is, for each of 16 rounds, the participant was asked to choose between two options. The attributes (e.g., what to smoke, place to smoke, etc.) remained the same for each choice set, but the values those attributes took (e.g., cigarettes vs. hookah, okay place vs. unpleasant place) varied each round. Thus, the participants were always choosing between two options based on a set list of attributes, but the value or label of the attribute changed across rounds.

**Table 1:** Discrete choice experiment attributes and levels

Feature (*Attribute*)	Options (*Levels*)				
1. What are you smoking	Cigarettes	Cigars	Hookahs	E-cigarettes	
2. The place where you can smoke and the people who are around you	Pleasant	Okay	Uncomfortable		
3. Kick or satisfaction from smoking	Very bad	Somewhat bad	Neutral	Somewhat good	Very good
4. Cost of the smoking	No cost (free)	$ 0.25 per smoke	$ 0.50 per smoke	$ 1.50 per smoke	$ 5.00 per smoke
5. Addictiveness	Not at all addictive/able to quit at any time	Moderately addictive/would be somewhat difficult to quit		Highly addictive/would be very difficult to quit	
6. The smell of smoke	Pleasant smell that quickly goes away	Odorless, no smell	Strong odor that lingers and stays on clothes		
7. Long-term health risks	No or minimal long-term health risks	Some chance (20%) of serious illness in the future	Moderate chance (60%) of serious illness in the future	High chance (90%) of serious illness in the future

The levels for each attribute were determined using feedback from the interviews, the trial, and the existing literature. The price of cigarettes and cigars was marginal price per use (i.e., price for a pack of cigarettes or for a single cigar). However, for hookahs and e-cigarettes, consumers must purchase the tobacco dose or a kit, which includes a battery package and a charger, and buy bottles of e-cigarette liquid. Participants were told to assume that the price of each unit was the marginal price of the use.

Sawtooth Software (v 8.2.4) was used to create a total of 16 choice set designs, each with seven attributes with a D-optimal design and determining the minimum sample size. Ten versions of the survey were used, and the efficiency of the design was 96.4% based on the geometric mean of the eigenvalues, 69.2% for the maximum standard error for prediction over the candidate set and 92.6% based on the algebraic mean of the eigenvalues.

### Measures definitions, attributes and levels

The definitions of people who smoke, people who vape, and nonsmokers are based on adult tobacco use nomenclature found in the CDC’s National Center of Health Statistics [30], the World Health Organization Global Adult Tobacco Surveillance System [31], and consistent with studies assessing U.S. tobacco use prevalence estimates [32, 33] using a people-first language approach [34].

People who smoke were identified as people who had smoked any product (cigarette, cigar, or hookah) more than 100 times in their lifetime and smoked every day in the last 30 days, and people who vape were identified as people who had vaped more than 100 times in their lifetime and vaped every day in the last 30 days. However, analysis of the responses suggested that very few people who vape met the criteria of a smoker as well, suggesting that they should more appropriately be viewed as ‘dual use’. Based on this, the categories were determined by:

- *Nonsmokers*: Persons who do not use any tobacco products as self-reported having never smoked or vaped in the last 30 days and never smoked or vaped more than 100 times in their lifetime.
- *People who vape*: Persons who vaped e-cigarettes 100 or more times in their lifetime and vaped e-cigarettes in the past 30 days.
- *People who smoke* *(cigarettes, cigars and/or hookahs)*: Persons who have use any combustible tobacco products 100 or more times in their lifetime, smoked one or more of the products in the past 30 days, and were not a person who vape e-cigarettes.

### Perceptions of vaping and smoking

Participants were asked their perceptions of smoking, including the cost for a pack of cigarettes, the place where they would most likely smoke, the kick they would receive, the addictiveness, the smell, and the health risk using the categories shown in Table 1. The respondents were then asked the same questions regarding their perceptions of vaping.

The Qualtrics distribution service was employed to reach a national survey audience and conduct their own participant verification and authentication to prevent web bot threats. A screening survey was used to identify an equal number of ‘people who smoke/people who vape’ and nonsmokers using the questions “Have you vaped or smoked any product (e.g., cigarettes, e-cigs, cigars, or hookahs) more than 100 times in your lifetime?” and then “Have you vaped or smoked any product (e.g., cigarettes, e-cigs, cigars, or hookahs) in the last 30 days?”. Respondents who answered ‘yes’ to both were assumed to be a person who smokes/person who vapes. At the conclusion of the survey, participants were asked for demographic information. The survey was available in English only. At the end of the survey distribution, the verified panel data did not contain missing data. The study was approved by the IRB Board at the University of California, Merced. Informed consent was obtained from all participants before the start of the survey.

## Data analyses

### Discrete choice analysis

The DCE choices were analyzed using conditional logit model (CLM) [35]. The attributes for cost and health risk were linearized. The results were analyzed for the entire sample and subsequently by smoking status.

Marginal probabilities of the choice between vaping and smoking were calculated using the perceptions of vaping and smoking. That is, for the entire sample and then for each group (people who smoke, people who vape, and nonsmokers) separately, the probability of each response (e.g., “pleasant place to smoke, okay place to smoke, or uncomfortable place to smoke”) was multiplied by the corresponding coefficient from the CLM and summed to estimate the utilities for each option. The marginal probabilities were then determined according to:

$$\mathrm{MP}(\mathrm{Vi}) =\mathrm{ exp}({\mathrm{Uv}}_{\mathrm{i}})/(\mathrm{exp}({\mathrm{Uv}}_{\mathrm{i}}) +\mathrm{ exp}({\mathrm{Us}}_{\mathrm{i}}))$$

where MP(V_i_) is the probability of vaping for group i, Uv_i_ is the utility associated with vaping for group i, and Us_i_ is the utility associated with smoking for group i. The probability of smoking for group i was 1 – MP(V_i_).

Finally, the marginal probabilities were calculated for several policy options aimed at promoting vaping. The base case was calculated using the strengths of preferences (coefficient values) from the DCEs (both combined for the entire sample and for each group separately) and the perceptions of each attribute (e.g., whether it was a pleasant, neutral or unpleasant place to smoke or vape). Thus, the base case represents the probability of choosing either to vape or to smoke given their perceptions of each product. The ‘base case’ to which these policies were compared used their perceived values of each of the different attributes, with the final utility being the weighted average of the responses:

$${\mathrm{Uv}}_{\mathrm{i}} = {\sum}_{\mathrm{j}}({\mathrm{\sum}}_{\mathrm{k}}({\mathrm{prob}}_{\mathrm{k}} * {\upbeta }_{\mathrm{k}}))$$

where j are the attributes, prob_k_ is the percentage who choose option k for attribute j, and β_k_ is the coefficient from the discrete choice results for group i corresponding to option k of attribute j. The base case analysis was computed in two ways: using the average price that each group reported as the cost of smoking or vaping and using a common price ($1) for each. Because each individual would pay a similar, market price, the base analysis with the common price is used for the comparisons of the impact of policy changes.

The policy options explored in the marginal analysis were as follows:

- Doubling the price of smoking,
- Requiring all smoking be conducted in an uncomfortable place,
- Creating a very bad kick from smoking,
- Emphasizing the high addictiveness of smoking, and
- Emphasizing the high health risk of smoking.

In addition, to identify the impact should policy makers consider increasing the price of vaping to discourage nonsmokers from vaping, the marginal analysis also explored the impact of the following:

Doubling the price of vaping.

## Results

### Sample

A total of 525 adults aged 18 to 88 years of age participated in the national survey. The mean age of the participants was 46 years old (± 16 years). Of the total sample, 52% were women, and 76% were Caucasian. Most of the participants were employed and had at least some college education. Table 2 includes the demographic characteristics of the study participants.

**Table 2:** Demographic summary of the survey participants and smoking group %

	All	Nonsmoker	People who vape	People who smoke
	*N* = 524	*n* = 343	*n* = 59	*n* = 143
Male	47.5%	43.3%	58.6%	52.4%
Female	52.5%	56.7%	41.4%	47.6%
African American	9.7%	8.4%	13.8%	11.2%
Asian/Pacific Islander	5.5%	7.1%	1.7%	3.5%
Hispanic/Latino	6.1%	6.2%	13.8%	2.8%
White/Other	80.9%	80.8%	74.1%	83.9%
18–29	19.3%	16.7%	27.6%	21.7%
30–49	38.2%	36.2%	50.0%	37.8%
50–64	24.4%	24.5%	19.0%	26.6%
65 +	18.1%	22.6%	3.4%	14.0%
High school or below	18.1%	12.7%	16.9%	30.3%
Some college/2-year degree	37.6%	37.5%	44.1%	34.5%
College degree or graduate	44.3%	49.8%	37.3%	33.8%
People who vape only			16.9%	
People who vape and smoke			83.1%	

### Discrete choice results: entire sample

The results from the conditional logit analysis are shown in Table 3. The first model (Model 1) provides the coefficient results for each attribute included in the experiment. The second model (Model 2) reports the results with cost and health risk linearized. As expected, all results had negative slopes with different degrees of magnitude. The results suggest that cigars and hookahs were the least preferred and that individuals were averse to uncomfortable places to smoke, very bad kicks, highly addictive tobacco products, strong odors, and tobacco products with high health risks.

**Table 3:** Conditional and linearized models for all participants and by smoking status

	Model 1	Model 2	Model 3		
	Conditional	Linearized	Nonsmokers	People who vape	People who smoke
	$$\upbeta$$ $$\text{(SE)}$$	$$\upbeta$$ $$\text{(SE)}$$	$$\upbeta$$ $$\text{(SE)}$$	$$\upbeta$$ $$\text{(SE)}$$	$$\upbeta$$ $$\text{(SE)}$$
Type of Product					
Cigarette	*-*	*-*	*-*	*-*	*-*
Cigar	-.314***	-.312 ***	0.020*	-0.445	-0.969*
	(-0.04)	(-0.04)	(-0.05)	(-0.12)	(-0.08)
Hookah	-.313***	-.303 ***	0.026**	-0.205	-1.059*
	(-0.04)	(-0.04)	(-0.05)	(-0.12)	(-0.08)
E-cigarette	-0.029	-0.026	0.188*	0.326	-0.59*
	(-0.04)	(-0.04)	(-0.05)	(-0.12)	(-0.08)
Place to Smoke					
Pleasant	-0.029	-0.028	0.022	-0.089	-0.093
	(-0.03)	-0.03)	(-0.04)	(-0.10)	(-0.06)
Okay	*-*	*-*	*-*	*-*	*-*
Uncomfortable	-.104**	-.110**	-0.029*	-0.318*	-0.204*
	(-0.03)	(-0.03)	(-0.05)	(-0.11)	(-0.07)
Kick or Satisfaction					
Very bad	-.163***	-.160***	-0.15**	0.101	-0.29*
	(-0.04)	(-0.04)	(-0.06)	(-0.13)	(-0.09)
Somewhat bad	-.140**	-.145**	-0.138**	-0.01	-0.221*
	(-0.05)	(-0.04)	(-0.06)	(-0.14)	(-0.09)
Neutral	*-*	*-*	*-*	*-*	*-*
Somewhat good	0.032	0.023	0.025	0.327**	-0.063
	(-0.05)	(-0.05)	(-0.06)	(-0.15)	(-0.1)
Very good	0.035	0.027	0.044	0.206	-0.05
	(-0.05)	(-0.04)	(-0.06)	(-0.14)	(-0.09)
Cost					
No cost (free)	*-*				
$0.25	-0.078				
	(-0.05)				
$0.50	-0.065				
	(-0.05)				
$1.50	-.231***				
	(-0.04)				
$5.00	-.569***				
	(-0.05)				
Linear	*-*	-.109***	-0.119*	-0.082*	-0.12*
		(0.00)	(-0.01)	(-0.02)	(-0.01)
Addictiveness					
Not at all addictive	*-*	*-*	*-*	*-*	*-*
Moderately addictive	-0.053	-0.053	-0.106*	0.12	-0.031
	(-0.03)	(-0.03)	(-0.04)	(-0.10)	(-0.06)
Highly addictive	-.201***	-.204***	-0.327**	0.027	-0.116
	(-0.03)	(-0.03)	(-0.05)	(-0.10)	(-0.07)
Smell					
Pleasant	-0.017	-0.019	-0.089	0.074	0.068
	(-0.03)	(-0.03)	(-0.04)	(-0.10)	(-0.06)
Odorless	*-*	*-*	*-*	*-*	*-*
Strong odor that lingers	-.175***	-.171***	-0.225*	-0.194**	-0.09
	(-0.03)	(-0.03)	(-0.04)	(-0.10)	(-0.06)
Long-term Health Risks					
No or minimal	*-*				
Some chance (20%)	-.286***				
	(-0.04)				
Moderate chance (60%)	-.918***				
	(-0.04)				
High chance (90%)	-1.33***				
	(-0.46)				
Linear	*-*	-.014***	-0.02*	-0.006*	-0.01*
		(0.001)	(0.01)	(0.001)	(0.01)

Levels of significance: *p < .05; **p < .01; ***p < .001

### Smoking status

As shown in Table 2, people who smoke and people who vape in the sample tend to be younger (average age 43 and 40, respectively) relative to nonsmokers (average age 50) and male (53% and 58% male, respectively) compared to nonsmokers (42% male). Most people who vape (83%) were also people who smoke, with only 17% reporting that they only vaped and did not smoke any tobacco product. Table 4 reports the results from their perceptions of cigarette smoking and vaping. Overall, views of the benefits of smoking or vaping (i.e., the kick or satisfaction) were higher for people who smoke and people who vape than for nonsmokers. People who vape rated the kick from vaping (62%) and from cigarettes (64%) as good or very good. Fifty-six percent of people who smoke, in contrast, rated the kick from smoking as good or very good, but only 26% rated the kick from vaping as good or very good. This is consistent with the result that people who vape in our sample tended to be both people who vape and people who smoke, whereas people who smoke only used tobacco products.

**Table 4:** Perceptions of smoking and vaping

	Cigarettes				Vaping			
	All	Nonsmokers	People who vape	People who smoke	All	Nonsmokers	People who vape	People who smoke
Cost per cig or vape*	$.45	$.39	$.67	$.49	$.80	$.70	$1.12	$.88
Place								
Pleasant	53%	48%	71%	58%	47%	44%	67%	46%
Okay	30%	27%	26%	38%	36%	34%	29%	43%
Uncomfortable	17%	25%	3%	4%	17%	22%	3%	11%
Kick								
Very bad	33%	49%	12%	4%	24%	32%	3%	15%
Bad	9%	9%	5%	10%	11%	13%	10%	8%
Neutral	26%	25%	19%	31%	42%	41%	24%	51%
Good	21%	12%	36%	34%	15%	12%	26%	18%
Very good	12%	5%	28%	22%	7%	1%	36%	8%
Addictiveness								
Not at all	10%	11%	7%	10%	19%	15%	17%	29%
Moderate	24%	18%	23%	34%	50%	47%	62%	48%
Highly	67%	74%	71%	56%	32%	38%	21%	22%
Smell								
Pleasant	15%	8%	33%	25%	28%	19%	57%	35%
No smell	9%	4%	7%	20%	47%	49%	33%	48%
Strong smell	76%	88%	60%	57%	25%	32%	10%	15%
Long term health risks								
No risk	4%	5%	2%	6%	15%	11%	17%	22%
Some (20%)	10%	3%	12%	25%	31%	24%	45%	41%
Moderate (60%)	29%	25%	38%	34%	29%	31%	29%	24%
High (90%)	57%	67%	48%	36%	25%	33%	9%	12%

^*^Assumes 1 ml per vape

The other notable difference between the groups was their assessment of the long-term health risk and addictiveness of each product. Seventy-four percent of nonsmokers and 71% of people who vape rated cigarettes as highly addictive, compared with 56% of people who smoke. All groups rated vaping as less addictive, with people who vape (21%) and people who smoke (22%) having lower ratings than nonsmokers (38% rated as highly addictive). While this same pattern held for health risk, with 92% of the nonsmokers rating the long-term health risk as moderate or high compared with 86% of people who vape and 69% of people who smoke, all groups rated the health risk of vaping as being lower (64% for nonsmokers, 38% for people who vape, and 36% for people who smoke).

### Discrete choice results: by smoking status

The results from the conditional logit analysis by smoking status are shown in Table 3. People who smoke and people who vape stated cigars (β = -0.969 *p* < 0.05) and hookahs (β = -1.059 *p* < 0.05) as their least preferred tobacco smoking option, with people who vape having a strong preference for e-cigarettes when compared with cigarettes, and people who smoke showing a strong preference for cigarettes. Nonsmokers reported an affinity for e-cigarettes (β = 0.188 *p* < 0.05) compared with cigarettes. All groups reported a significant aversion to uncomfortable places (β = -0.318 *p* < 0.05) to smoke or vape and people who smoke were also less likely to favor products that had a somewhat bad kick (β = -0.221 *p* < 0.05). People who vape, on the other hand, generally did not report the kick as being an important consideration. Nevertheless, a somewhat good kick was their preferred option (β = 0.327 *p* < 0.01).

All groups – people who smoke, people who vape, and nonsmokers – reported cost (β = -0.12 *p* < 0.05) as being a significant factor, but only nonsmokers reported the addictiveness (β = -0.327 *p* < 0.01) and the smell (β = -0.225 *p* < 0.05) of the product as being a significant concern. People who smoke and nonsmokers disliked a very bad (β = -0.29 *p* < 0.01) or somewhat bad kick (β = -0.138 *p* < 0.05) or satisfaction. People who smoke and nonsmokers are also more concerned with cost (β = -0.119 *p* < 0.05) compared to people who vape. While all three groups were significantly concerned with long-term health risks, the relative importance was stronger among nonsmokers (β = -0.02 *p* < 0.05).

### Marginal analysis

The results suggest that all groups were sensitive to place to smoke/vape, price, and long-term health risks. The marginal analysis shown in Table 5 shows the marginal probabilities for a choice between cigarette smoking and vaping under a variety of conditions. The marginal analysis uses the results from the linear DCEs reported in Tables 3 and 5 and the perceptions of smoking and vaping reported in Table 4.

**Table 5:** Marginal probabilities

	All		Nonsmokers		People who vape		People who smoke	
	Cig	Vape	Cig	Vape	Cig	Vape	Cig	Vape
Base case	48%	52%	41%	59%	42%	58%	64%	36%
Base case—common price	38%	62%	30%	70%	36%	64%	58%	42%
Double cost of smoking	36%	64%	27%	73%	35%	65%	55%	45%
Force smoking into uncomfortable place	36%	64%	29%	71%	31%	69%	54%	46%
Very bad kick from smoking	36%	64%	29%	71%	38%	62%	52%	48%
Emphasize addictiveness of smoking	37%	63%	28%	72%	36%	64%	55%	45%
Emphasize health risk of smoking 90%	32%	68%	24%	76%	33%	67%	50%	50%
Emphasize health risk of smoking 20%	57%	43%	56%	44%	44%	56%	66%	34%
Doubling the price of vaping	41%	59%	32%	68%	38%	62%	61%	39%

The results suggest that for the base case analysis using the common price of smoking and vaping, the probability of the participants choosing vaping over cigarette smoking was 62% overall, including 64% of people who vape and 70% of nonsmokers. This is consistent with the nonsmokers having a more negative view, including higher health concerns, about smoking than vaping. People who smoke were more likely (58%) to choose cigarettes over vaping.

Table 5 shows the impact on smoking of doubling the price of cigarettes, regulating cigarette smoking to be done in an uncomfortable location, reducing the kick from cigarette smoking, and emphasizing the addictiveness of the cigarettes. The results suggest that these policies had only a modest impact on the behavior of either people who smoke or people who vape, increasing the probability of vaping from between 1 and 6%. Emphasizing the health risks of smoking to 90% reduces the probability of using cigarettes by 8% for people who smoke and nonsmokers but has a relatively smaller impact on people who vape (3% reduction). However, nonsmokers were very sensitive to changes in the risks associated with smoking, with the probability of their smoking cigarettes increasing by 32% when the health risk was 20% compared with 90%. Finally, the results suggest that doubling the price of vaping would increase the probability of cigarette smoking only marginally, ranging from 3% for people who smoke and nonsmokers to 2% for people who vape.

## Discussion

This study sought to estimate the potential impact of policies aimed at promoting vaping among people who smoke. The results suggest that, overall, price increases are likely to have only relatively modest success in moving people who smoke to start vaping or encouraging people who vape (the majority of whom also smoke tobacco products) to vape rather than use cigarettes. The study also sought to understand how changing the price of vaping would impact the perceptions of nonsmokers toward smoking. The results suggest that nonsmokers view vaping more positively than smoking, are not very sensitive to price changes, but are sensitive to the health impacts.

The results of this study are consistent with the findings of previous DCE and revealed preference studies examining the interaction between smoking and vaping. As with other studies, nonsmokers in this study stated a positive preference for the option of smoking e-cigarettes [13, 20]. This includes the findings that people who vape are sensitive to changes in the place and conditions of vaping [36], that vaping prices can influence demand for vaping [18] and smoking, and that people who vape and people who smoke are sensitive to the health risk [37]. There is also support for the finding that many adult people who vape also use tobacco products [12]. The marginal analysis suggests that people who smoke (who do not report vaping) are relatively favorable toward vaping is consistent with previous studies suggesting that people who smoke might fail to recognize the benefits of vaping given their preferences [16].

The study adds to the literature by examining the preferences of people who smoke, people who vape, and nonsmokers together and separately to estimate the likely impacts of policy changes aimed at promoting vaping. The results suggesting that people who smoke are more attentive to a negative kick rather than a positive kick are consistent with the finding from behavioral economics that people who smoke may be more motivated to avoid a negative outcome or have relief from a negative state than obtain a positive state [38–40]. People who vape, on the other hand, reported a slightly greater preference for a positive kick. This might occur because the people who vape generally reported higher rates of the kick from vaping (66%) and smoking (64%) as being good or very good than the people who smoke reported the kick from vaping (37%) or smoking (56%). Finally, the findings suggesting that people who smoke and people who vape had less concern about the long-term health risk are consistent with the rational view of addiction [41].

The implications of these results for policy makers and others seeking to promote people who smoke to change from smoking to vaping are less reassuring. The results suggest that increasing the price of cigarettes or requiring smoking to be conducted in an uncomfortable place would likely result in only a limited increase in the probability of people who smoke switching to vaping. Changing the amount of nicotine or flavor (kick) associated with smoking would increase the probability of vaping slightly and have little impact on nonsmokers or vaping preferences, but the most significant change would come from increasing the perceptions of the risk of smoking. However, even these results are likely to be muted because most nonsmokers (94%), people who vape (86%), and people who smoke (70%) already see cigarettes as having moderate or high health risks. Increased awareness of the risks of vaping, on the other hand, is likely to have larger impacts in switching from vaping to cigarettes because a smaller majority of nonsmokers (64%) and only a minority of people who vape (38%) and people who smoke (36%) see vaping as having moderate or high health risks.

These findings are particularly important following the announcement of the FDA to restrict nicotine levels [42, 43].

This study has several limitations. First, the participant sample was predominately white, with only a small number of participants identifying groups that the CDC reports as having the highest rates of tobacco use. Among people who smoke and/or people who vape we did not inquire about past attempts to quit or variations in the frequency of tobacco product use, this may have had affected the study’s results. The sample is also not representative of the wider population in that it relied on an online survey, therefore limiting the participation further. Thus, caution should be used in drawing conclusions from this study for the wider population.

Second, a key choice in developing a DCE is whether to include an ‘opt-out’ in the choice sets or to force respondents to select one of the options. The decision of whether it is reasonable to include or exclude an opt-out option is context specific [see 12 for a thorough discussion], with the opt-out allowing participants to indicate that they would not prefer either choice. In the present context, interviews with nonsmokers suggested that they would predominantly choose the opt-out option if provided. Because of the interest in comparing the preferences of people who smoke and people who vape to those of nonsmokers, the opt-out option was not offered. Additionally, perception questions followed the conclusion of the DCE choice sets.

Last, DCEs are studies based on stated survey responses and caution should be used in drawing policy conclusions, particularly if a study is poorly designed [44]. While the results increase the body of knowledge available to public health professionals concerned with developing evidence-based patient and community-centered interventions to address current tobacco use and prevention, further evidence is needed from revealed preference studies to confirm these results.

## Data Availability

Data available on request. The data underlying this article will be shared on reasonable request to the corresponding author.
